# Consumers’ expected information search behavior for a pharmaceutical treatment

**DOI:** 10.3389/fphar.2025.1466352

**Published:** 2025-02-13

**Authors:** Yifei Liu, Jack E. Fincham, Morgan L. Sperry

**Affiliations:** ^1^ Division of Pharmacy Practice and Administration, University of Missouri – Kansas City School of Pharmacy, Kansas City, MO, United States; ^2^ Osher Life Long Learning Institute, The University of Arizona, Tucson, AZ, United States

**Keywords:** information search, information seeking, consumer behavior, health information, pharmaceutical treatment, medication

## Abstract

**Objective:**

The Comprehensive Model of Information Seeking (CMIS) integrates multiple factors influencing information seeking. This study was to identify factors impacting consumers’ expected information search behavior for a pharmaceutical treatment. By examining the predictive utility of these factors, the CMIS could be improved in the context of health information search.

**Methods:**

A telephone interview was administered to a random-digit-dialed sample of 2,186 adult residents in a southern U.S. state. Measurements included expected information search for a pharmaceutical treatment within the next 2 weeks, self-rated health (SRH), extraversion, and demographics. SRH was measured on a 4-point scale (Excellent-4, Poor-1), with higher scores indicating better subjective health. A logistic regression was conducted, in which the outcome variable was the expected information search for a pharmaceutical treatment, and covariates were SRH, extraversion, and demographics.

**Results:**

A total of 505 individuals participated. On average, they were 57 years old, and 61% of them were female. Fourteen percent of them expected to seek information for a pharmaceutical treatment. The logistic regression was significant (p < 0.01). SRH was a significant negative predictor (p < 0.05) and gender (female) was a significant positive predictor for the expected information-seeking behavior (p < 0.05).

**Conclusion:**

Consumers with lower SRH and women were more likely to perform expected information search for a pharmaceutical treatment. These findings have implications for both the modification of the CMIS and the provision of healthcare interventions.

## Introduction

Consumers have become more proactive in seeking health information, driven by the growth of the Internet, the rise of social media, and the proliferation of information (Hesse et al., 2005; Fox and Duggan, 2013; Chen and Wang, 2021). Overall, there can be six domains of health-related information “diet, exercise, illness or disease, medications, parenting, and treatments” (Weaver et al., 2010). The domain of medications (i.e., pharmaceutical treatments) is important, because understanding how consumers search for information in this domain is essential for stakeholders including healthcare professionals, policymakers, and pharmaceutical manufacturers. Stakeholders can better meet the needs of consumers to improve health outcomes and make healthcare delivery more efficient. On one hand, stakeholders can create more relevant and understandable resources for patient education after learning about common queries and concerns about a pharmaceutical treatment. This can lead to health literacy and empower patients to make informed decisions about their health (Bhattad and Pacifico, 2022). On the other hand, misinformation may stem from various sources and cause public health issues such as vaccine hesitancy (Lewandowsky et al., 2012; Loomba et al., 2021). By recognizing the patterns in how consumers seek and interpret information, stakeholders can develop strategies to combat misinformation and address barriers to accessing specific pharmaceutical treatments.

Understanding how consumers seek health information for pharmaceutical treatments, requires a structured framework. The Comprehensive Model of Information Seeking (CMIS) offers such a framework, as it integrates multiple factors influencing how and why people seek information (Johnson and Meischke, 1993; Johnson et al., 1995). According to the CMIS, information-seeking actions are impacted by information carrier factors such as characteristics and utilities, and utilities are affected by antecedents such as demographics, experience, salience, and beliefs. The characteristics of information carriers refer to their credibility and accuracy of the information, and the utilities of information carriers relate to the relevance and usefulness of the information in achieving one’s goals (Johnson, 1997; Johnson et al., 2001). Although widely used, a meta-analysis reveals that the original CMIS lacks an acceptable model fit (Yang et al., 2023). In the context of searching for health information, we believe that there are three areas that are worth exploring to improve the CMIS. First, the model does not include self-rated health (SRH), an individual’s own assessment of his or her health. SRH has since been used as a single-item question to rate one’s own health from “poor” to “excellent” (Krause and Jay, 1994; Idler and Benyamini, 1997). Second, the model does not specify the impact of personality traits. One example is that extroverts are more likely to engage in active information seeking (Heinström, 2003). Third, whether antecedents are in fact mediated by utilities remains unclear, since it is reported demographics directly affect online health information search (Jia et al., 2021).

We hypothesized that one’s SRH may drive the expected information search for a pharmaceutical treatment. In addition, demographics and extraversion, a personality trait involving the sociability, talkativeness, and assertiveness of a person (McCabe and Fleeson, 2012), may directly impact the information search. The objective of this study was to identify factors impacting consumers’ expected information search for a pharmaceutical treatment. By examining the predictive utility of these factors, the CMIS could be improved in the context of health information search.

## Methods

We contracted with a survey research center (SRC) in a southern U.S. state to conduct a telephone interview. The interview was incorporated as part of a larger telephone interview administered by the SRC to a random-digit-dialed sample of 2,186 adult residents in that state. The SRC estimated the sample size using a formula: Standard error = square root of (P*Q)/n, where P = the proportion of the population exhibiting a characteristic; Q = (1-P), the proportion not exhibiting the characteristic; and n = sample size. The SRC anticipated a total of 505 complete telephone interviews, and the sampling method aimed to ensure that every adult resident within the sample had an equal probability of being selected for participation. This provision of equal opportunity of selection was necessary for a probability sample to be obtained. The estimated sample size was subject to sampling error of ± 4.4% at the 95% confidence interval. Measurements included the expected information search for a pharmaceutical treatment within the next 2 weeks (yes/no for each behavior, a dichotomous/nominal variable), SRH (4-point scale, considered as an interval variable), extraversion (3 items, 5-point scale, an interval variable), and demographics. The demographics consisted of age (a ratio variable), race (Caucasian/non-Caucasian, a dichotomous/nominal variable), gender (male/female, a dichotomous/nominal variable), marital status (married or not, a dichotomous/nominal variable), education (three categories, an ordinal variable), and metropolitan statistical area (yes/no, a dichotomous/nominal variable). The measurement for SRH was to ask people to rate their health as excellent, good, fair, or poor (Krause and Jay, 1994; Idler and Benyamini, 1997). Acknowledging that the SRH measure likely reflected an underlying continuous concept of subjective health (Kananen et al., 2021; Hamplová et al., 2022), we treated SRH as an interval variable. To ensure clarity in data analyses, we scaled the measure from “Poor-1” to “Excellent-4”, with higher scores indicating better subjective health. The 3-item measurement for extraversion were selected or modified from previous instruments (John and Srivastava, 1999). Below are the measurements except demographics.

SRH: “Please describe your overall health status.” (Excellent-4, Good-3, Fair-2, Poor-1).
Expected information seeking for a pharmaceutical treatment: “Will you search for information about a pharmaceutical treatment for such medical condition within the next 2 weeks?” (Yes/No).
Extraversion (strongly agree-5, strongly disagree-1): (1) “I see myself as someone who is outgoing.“; (2) “I see myself as someone who is talkative”; (3) “I see myself as someone who generates a lot of enthusiasm.”

Before conducting the interview, telephone interviewers participated in two comprehensive training sessions, each lasting 3 hours. These sessions encompassed a range of topics, such as methodologies, standard procedures for telephone interviewing, the interview’s objectives, a detailed elucidation of the instrument, and a practical exercise. During the telephone interview, upon locating a respondent and securing cooperation, quality-control procedures were implemented to ensure high-quality data collection. Supervisors were designated to oversee the interviewers and monitored approximately one-fifth to one-quarter of all interviews. This oversight helped to identify and rectify any interviewer errors.

Reliability analysis was performed for extraversion. A logistic regression was conducted, in which the outcome variable was the expected information search for a pharmaceutical treatment, and covariates were SRH, extraversion, and demographics. In the regression, extraversion was represented by taking the average of the 3 items for each participant. A series of statistical tests and procedures were conducted to verify the assumptions of a logistic regression, including the independence of errors, absence of multicollinearity, absence of outliers, and linearity between continuous covariates and the logit of the outcome variable. In addition, the Hosmer and Lemeshow Test was used to evaluate the fit of the logistic regression. The study was approved by the Institutional Review Board at the University of Georgia.

## Results

Of the 2,186 eligible respondents contacted, 505 yielded complete interviews. The average age of participants was 56.8 years old, the average SRH score was 3 on a 4-point scale, and the average score of extraversion measures was 3.74 on a 5-point scale (Table 1). Sixty-one percent of participants were female, 72% were Caucasian, 65% were married, 23% had a bachelor’s degree, and 76% resided in a metropolitan statistical area. Fourteen percent of them expected to seek information for a pharmaceutical treatment. The Cronbach’s alpha for 3 items of extraversion measures was 0.75, indicating high reliability.

**TABLE 1:** Participants’ characteristics.

Variables	Mean ± SD (range) or % (n)^a^	Total N^b^
SRH	3.04 ± 0.81 (1–4)	502
Excellent (4)	29.5% (148)	
Good (3)	50.6% (254)	
Fair (2)	14.3% (72)	
Poor (1)	5.6% (28)	
Extraversion	3.74 ± 0.79 (1–5)	487
Age	56.80 ± 18.41 (18–98)	497
Expected information search for a pharmaceutical treatment (Yes)	14.3% (68)	477
Gender (Female)	61.5% (305)	496
Race (Caucasian)	72.3% (353)	488
Marital status (Married)	64.8% (313)	483
Education		487
Below Bachelor’s degree	58.1% (283)	
Bachelor’s degree	22.7% (111)	
Above Bachelor’s degree	19.1% (93)	
Metropolitan Statistical Area (Yes)	76.4% (386)	505

Note: N varied due to missing data.

^a^ Mean and standard deviation were calculated for continuous variables. Frequency was calculated for categorical variables.

^b^ Total N varied due to missing data.

The logistic regression of expected information search for a pharmaceutical treatment passed the statistical tests or procedures of verifying the assumptions of a logistic regression. The regression was significant (p < 0.01) (Table 2). The significance of the Hosmer and Lemeshow Test was greater than 0.05, indicating a good fit. SRH was a significant negative predictor for the information-seeking behavior (p < 0.05), with an odds ratio of 0.70 (95% CI: 0.49, 0.98). In other words, an individual with lower SRH was more likely than an individual with higher SRH to perform expected information search for a pharmaceutical treatment. Gender (female) was a significant positive predictor for the expected information-seeking behavior (p < 0.01), with an odds ratio of 2.40 (95% CI: 1.26, 4.57). That is, compared with male consumers, female consumers were more likely to perform expected information search. Extraversion and other demographic variables were not significant predictors.

**TABLE 2:** Logistic regression model of expected information search behavior.

Outcome variable Covariates	Expected information search for a pharmaceutical treatment (yes/No) ** N = 447	
	Beta coefficient	Adjusted odds ratio (95% confidence interval)
SRH	−0.36*	0.70 (0.49, 0.99)
Extraversion	0.04	1.04 (0.72, 1.50)
Age	−0.01	0.99 (0.98, 1.01)
Race (Caucasian)^a^	−0.08	0.93 (0.49, 1.76)
Gender (Female)^b^	0.88**	2.40 (1.26, 4.57)
Marital status (Married)^c^	−0.07	0.94 (0.52, 1.70)
Education^d^		
Bachelor’s degree	0.32	1.38 (0.71, 2.67)
Above Bachelor’s degree	−0.87	0.42 (0.16, 1.13)
Metropolitan statistical area (Yes)^e^	−0.55	0.58 (0.31, 1.06)

Note: N varied due to missing data. ** Significant at the 0.01 level; * significant at the 0.05 level.

^a^ The reference group was non-Caucasian.

^b^ The reference group was male.

^c^ The reference group was non-married.

^d^ The reference group was those whose education level were less than bachelor’s degree.

^e^ The reference group was those who were not in a metropolitan statistical area.

## Discussion

This study was distinctive in its integration of SRH as a direct predictor of an information-seeking behavior, proposing that individuals’ subjective evaluations of their own health status can be as critical as other factors traditionally highlighted by the CMIS. Additionally, it establishes gender as a key determinant, underscoring that women may require interventions specifically tailored to improve medication use. Conversely, the non-significance of extraversion and other demographics in predicting information seeking for pharmaceutical treatments may imply the importance of perceived health needs and accessibility of information sources.

First, the study identified SRH as a significant negative predictor of expected information-seeking behavior for a pharmaceutical treatment. Although it is a subjective measure, SRH is associated with objective health outcomes such as mortality (DeSalvo et al., 2006). In this study, individuals who rated their health as fair or below could perceive a strong need for information about a pharmaceutical treatment, driving them to seek information actively. Conversely, individuals with higher SRH might feel less urgency to seek health information because they perceived their health status as good or above. This may result in reduced engagement with health information. One implication is that those with lower SRH could be targeted by health interventions to be more proactive in managing their health, including seeking information about pharmaceutical treatments to ensure proper use and adherence. Moreover, this study demonstrates a direct impact of SRH on the expected behavior, indicating that SRH should be placed in parallel with information carrier factors in the CMIS.

Second, gender was also found to have a direct impact on the expected behavior. This finding is consistent with the literature that demographics can directly influence information seeking (Jia et al., 2021). It has also been reported that women are more likely than men to seek online information for a diagnosis (Fox and Duggan, 2013). Women appear to be more likely to recognize symptoms and seek health information earlier than men. In addition, women often bear the primary responsibility for health in the family, leading them to seek more information to care for themselves and their loved ones. Understanding gender difference in information seeking can help customize interventions to improve medication use. For example, women-centered support groups can provide platforms for sharing information and experiences, promoting better medication use through peer support and collective learning. In the CMIS, demographics are a type of “antecedents” which needs to be mediated by “utilities” (Johnson and Meischke, 1993; Johnson et al., 1995). Our finding supports a direct relationship between gender and expected health information search.

**FIGURE 1:**
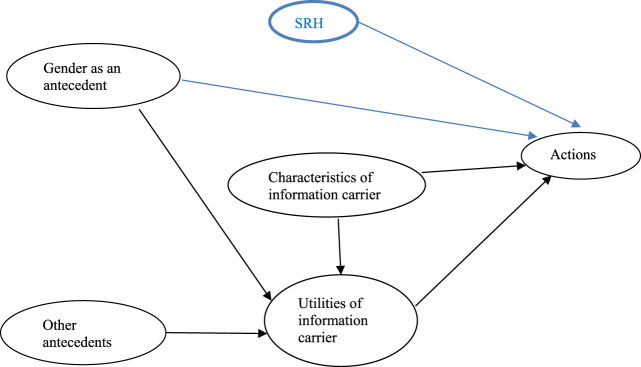
Proposed modifications of the CMIS for health information seeking. Note: Blue arrows and construct are proposed modifications.

Third, extraversion and the other demographics did not impact both the expected behavior. Extraversion seems to have minimal impact on information seeking for a pharmaceutical treatment. Our interpretation is that health information seeking is primarily driven by an individual’s perceived need for information. Consumers, whether introverted or extraverted, seek information for a pharmaceutical treatment when they feel it is necessary to manage their health conditions. Additionally, the increasing availability of health information through digital platforms has allowed individuals to seek information privately without the need for social interaction. This is particularly important for introverted individuals who may prefer to seek information in solitude. The convenience and anonymity of online resources may reduce the relevance of extraversion in health information seeking. Therefore, extraversion does not need to be added to the CMIS in the context of health information seeking. As for other demographics, they could be “antecedents” mediated through “utilities” in the CMIS without exerting any direct predictive power on the behavior. To summarize potential impact of our findings on the CMIS, we propose a modified model for health information seeking in Figure 1. The blue arrows represent added predictive relationships, while the black arrows represent original relationships. In addition, SRH is added as a new construct. Of note, the “actions” in this study were expected actions instead of actual actions.

Our findings should be viewed with a number of caveats. First, we measured an expected behavior within the next 2 weeks rather than the actual behavior, and used the expected behavior in the logistic regression. In the CMIS, expected behaviors were regarded as proxies for “actions”. However, there was no real temporal sequence between the covariates and the expected behavior, as they were measured at the same time. Second, this study was a portion of a larger telephone interview. Due to the limited time available in the larger telephone interview and to avoid response burden, we were unable to include all variables in the CMIS and test the full modified CMIS in Figure 1, such as whether gender or other demographics were mediated by “utilities”. For the same reason, we were only able to test the effect of extraversion, not other personality traits. Third, the sample was selected from adult residents in a southern U.S. state, and the generalizability of results is limited. Nevertheless, our study examined whether there was a direct effect of SRH, extraversion, and demographics including gender, on health information seeking. Future research could measure the actual information-seeking behavior and test the full modified CMIS. For instance, “characteristics of information carriers” is a domain worth exploring, particularly with the growing adoption of portals, health apps, and remote monitoring devices. Although research indicates that internet connectivity is generally linked to improved wellbeing (Vuorre and Przybylski, 2024), these digital tools have also raised significant access and privacy concerns, particularly among low-income, rural, and older populations (Sieck et al., 2021). In addition, our study reveals that the effect of gender (beta coefficient of 0.52) was greater than that of SRH for expected information seeking for a pharmaceutical treatment (beta coefficient of −0.36). Thus, another direction for future research is to understand why the prediction differences exist.

## Conclusion

A consumer with lower SRH was more likely to perform expected information search for a pharmaceutical treatment. Women were more likely to perform the expected information-seeking behavior. Extraversion and the other demographics did not impact the expected behavior. These findings have implications for both the modification of the CMIS and the provision of healthcare interventions.

## Data Availability

The original contributions presented in the study are included in the article, further inquiries can be directed to the corresponding author.
